# Gastric bezoar in a patient hospitalized in an eating disorder unit. Case report

**DOI:** 10.1192/j.eurpsy.2023.1802

**Published:** 2023-07-19

**Authors:** J. Torres Cortés, I. Esteban Avendaño, J. B. González del Valle, R. González Lucas, J. J. Padín Calo, J. P. Morillo González

**Affiliations:** 1 Psychiatry; 2 Family and Community Medicine, Hospital Universitario Ramón y Cajal, Madrid, Spain

## Abstract

**Introduction:**

It is well known that eating disorders are related to comorbidity. At least, half of these patients have other mental disorders and, in addition to it, the presence of physical comorbidity (cardiovascular, kidney, nervous system, digestive tract, metabolic or endocrine disorders) comes with a decline in life expectancy.

**Objectives:**

Description of a patient with a diagnosis of anorexia nervosa (AN) who developed a gastric bezoar during hospitalization.

**Methods:**

Case treated in a specific Eating Disorder Unit in a Third-Level Hospital.

**Results:**

26 years old woman with a diagnosis of AN hospitalized in General Psychiatric Unit with BMI of 11,78 kg/m2. Nasogastric tube was necessary and, after 1 month with a progressive weight recovery (BMI 13,84 kg/m2), the patient was transferred to the Eating Disorder Unit in order to follow specific psychological therapy. No incidence related to physical exploration or clinical analyses happened during this month apart from pancytopenia due to malnutrition.

However, 8 days after, patient developed nausea and had 3 vomit episodes, constant abdominal pain at hipogastrium (moderate intensity), dizziness, instability and constipation. The patient refused possibility of pregnancy. The physical exam showed bowel sounds augmented but no mass or peritoneal irritation appeared. Blood test results were normal. Abdominal X-Ray showed gastric dilatation with small bowel faeces sign, which suggested diagnosis of gastric bezoar.

The treatment was the dissolution of the bezoar by Coca-Cola, solving the symptoms completely.

The patient refused having eaten hair or any other kind of object or indigestible material but admitted to be following a strict vegan diet. Finally, after an endoscopy was done, the patient was diagnosed of phytobezoar.

**Conclusions:**

Based on literature, bezoars are rare in AN, being phytobezoars the most common between the types of bezoars. Nevertheless, there are some risk factors, such as delayed gastric emptying, dehydration or, in the case of phytobezoar, ingestion of food containing high amount of cellulose, hemi-cellulose, lignin, and tannins (celery, pumpkin, grape skins, prunes, raisins and, in particular, persimmons). Some of the symptoms caused by phytobezoar can be similar to those of the AN (abdominal pain, intestinal obstruction, poor appetite, vomiting, malnutrition, weight loss). Therefore, gastric bezoar could be an underdiagnosed or even undiagnosed disease in this group of patients. Taking this into account could reduce time until diagnosis and treatment, decreasing the risks associated.

**Disclosure of Interest:**

None Declared

